# The regulator of calcineurin 1 (RCAN1) inhibits nuclear factor kappaB signaling pathway and suppresses human malignant glioma cells growth

**DOI:** 10.18632/oncotarget.14479

**Published:** 2017-01-04

**Authors:** Xin Chen, Yuanyuan Hu, Shuo Wang, Xiulian Sun

**Affiliations:** ^1^ Department of Neurosurgery, Beijing Tiantan Hospital, Capital Medical University, Beijing, P. R. China; China National Clinical Research Center for Neurological Diseases, Beijing, P. R. China; Center of Stroke, Beijing Institute for Brain Disorders, Beijing, P. R. China; Beijing Key Laboratory of Translational Medicine for Cerebrovascular Diseases, Beijing, P. R. China; ^2^ Brain Research Institute, Qilu Hospital of Shandong University, Jinan, Shandong Province, China

**Keywords:** RCAN1, suppress, apoptosis, glioma, NF-κB signaling

## Abstract

Nuclear factor-kappaB (NF-κB) has a vital role in cell survival and inhibition of NF-κB had proven to be an efficient therapeutic pathway for various cancers though little is known about the underlying mechanism. Previously we identified regulator of calcineurin 1 (RCAN1) as an endogenous inhibitor of NF-κB signaling pathway in lymphoma. In the present study, we have solid data to show that RCAN1 can inhibit the nuclear translocation of NF-κB protein then affect the activity of NF-κB signaling pathway in glioma cells. Overexpression of RCAN1 markedly reduced glioma cells viability. We further found that the suppressing glioma cell growth was closely related to the pro-apoptosis effect, not by inhibiting proliferation by the arrest of cell cycle. Our study implicated a novel therapeutic approach for glioma by RCAN1 through inhibition of NF-κB signaling.

## INTRODUCTION

People with Alzheimer's disease (AD) may have lower risk of cancer [1], implying something elevated in AD can suppress cancer growth. Regulator of calcineurin 1 (RCAN1) is located on chromosome 21 in the region of 21q22.12. RCAN1 expression is elevated in the cortex of AD patients and overexpression of RCAN1 contributes to AD pathogenesis [2]. Recent studies show that RCAN1 can attenuate endothelial cell proliferation and angiogenesis [3], which contributes to malignant progression in most of the major forms of human cancer. RCAN1 gives rise to abroad cancer suppression via inhibiting the calcineurin pathway in the vascular endothelium in the transgenic mouse model of xenografted tumors [4]. A single extra copy of RCAN1 is sufficient to suppress tumor angiogenesis in mice [5]. Our recent reports showed that NF-κB signaling regulated RCAN1 isoform 4 gene transcription through a NF-κB responsive element [6] and RCAN1 as a novel inhibitor of NF-κB signaling pathway can suppress lymphoma growth in mice [7]. Meanwhile we find that overexpression of RCAN1 induced neuronal apoptosis [2]. But it is unknown if RCAN1 can suppress glioma growth.

Glioma, accounted for more than 70% of brain tumors, is the most common neoplasm of central nervous system [8]. According to the World Health Organization (WHO) classification, the most used glioma grading system, gliomas are subdivided to four grades (I–IV) based on the degree of malignancy [9, 10]. However, the understanding about the pathogenesis of glioma is still largely unclear and the prognosis of patients with malignant glioma is still poor despite combined treatments with surgery, chemotherapy, and irradiation [11, 12]. There thus is an urgent need to identify novel molecular markers as potential therapeutic targets for glioma.

Nuclear factor-κB (NF-κB) is a family of transcription factors, which is an crucial regulator of cell survival, proliferation and differentiation [13]. NF-κB signaling pathway has been considered as a therapeutic target in cancer because of its role in carcinogenesis [14]. Abnormal levels of constitutively activated NF-κB have been detected in various solid tumors [15], as well as glioma. We are interested to know if RCAN1 can affect NF-κB signaling and subsequent glioma tumorigenesis. In the present study, we showed that NF-κB was activated in human malignant glioma. RCAN1 inhibited NF-κB signaling activity in human glioma cells. Overexpression of RCAN1 markedly suppressed glioma cells’ viability and increased apoptosis. Our studies provided a potential therapeutic pathway for glioma by overexpressing RCAN1 to inhibit NF-κB signaling.

## RESULTS

### NF-κB signaling pathway proteins were elevated in glioma tissue

**Figure 1:**
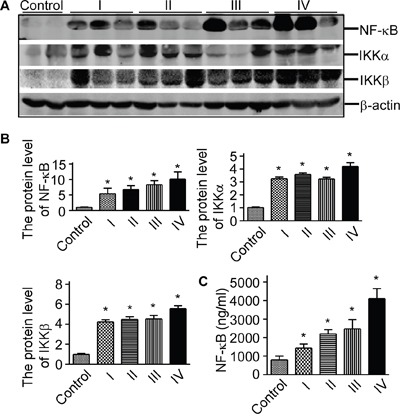
NF-κB signaling pathway proteins were elevated in glioma tissue **A**. Protein lysates from glioma tissues and controls were separated on a 10% glycine SDS-polyacrylamide gel. The lysates were immunoblotted with anti-NF-κB/p65, anti-IKKα, and anti-IKKβ at a dilution of 1:1000 (*lanes 1, 2* and *3*). β-actin was used as the internal control. **B**. Quantification of Western blots showed that NF-κB/p65, IKKα, and IKKβ were increased in glioma tissues. Values represent mean ± S.E. (*n* = 3), *p <0.05 by Student's *t* test. **C**. The expression of NF-κB in glioma was measured using ELISA assay. The values represent the means ± S.E. (n = 3),*p<0.05 by Student's t test.

To investigate the levels of NF-κB signaling pathway proteins in glioma, we extracted the proteins of glioma. NF-κB/p65, IKKα, and IKKβ western blot showed that NF-κB/p65, IKKα, IKKβ were significantly increased in glioma rather than the normal controls (*p*<0.05, Figure 1A and Figure 1B). To further characterize human NF-κB expression in glioma tissues, the exprssion of NF-κB in glioma was detected by human NF-κB p65 ELISA assay (Figure 1C, *p*<0.05). And the ELISA assay showed that the expression of NF-κB was increased in glioma tissue compared with the control. Our data suggested that NF-κB signaling pathway proteins were elevated in glioma tissue.

### RCAN1 suppressed cell viability in glioma cell lines

**Figure 2:**
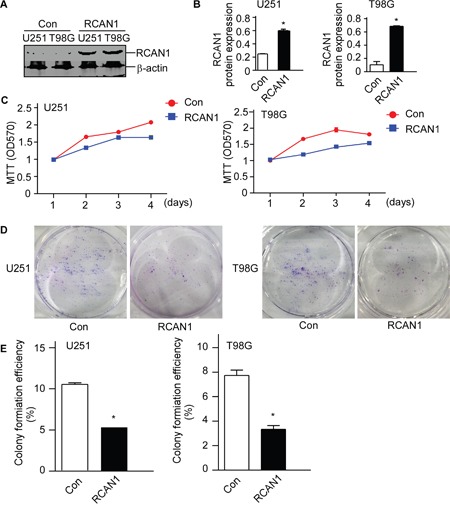
RCAN1 decreased cell viability in glioma cell lines U251 and T98G cells were infected with lentivirus expressing RCAN1 (RCAN1) or negative control (Control) for 72 hrs. **A**. Western blot was performed to assess the RCAN1 protein level. RCAN1 protein levels were normalized to β-actin. The results present the fold-increase relative to control. **B**. Quantification of (A). Values represent means ± SE (n = 3),*p <0.05 by Student's *t* test. **C**. U251 and T98G cells viability were measured by MTT assay. MTT cell viability was measured by the absorbance at 570 nm (ref: 670 nm) at indicated times after infecting 72 hrs. The values represent the means ± S.E. (n = 3), *p*<0.05 by two-way ANOVA. **D**. U251 and T98G cells viability were measured by cell colony formation assay. **E**. Quantification of (D). Values represent means ± SE (n = 3),*p <0.05 by Student's *t* test.

Previously, we showed RCAN1 inhibited NF-κB signaling in lymphoma. To investigate the increased NF-κB signaling in glioma were also associated with RCAN1 expressions. Western blot assay showed that lentivirus expressing RCAN1 could efficiently elevate RCAN1 protein levels to 0.601± 0.021 compared with 0.240±0.006 in U251 cells (*p*<0.05, Figure 2A and B), and 0.686±0.006 compared with 0.104±0.048 in T98G cells (*p*<0.05, Figure 2A and B). To examine the effects of RCAN1 on the viability of glioma cell line U251 and T98G cells, MTT assay and colony formation assay were performed after infecting with lentivirus expressing RCAN1 or negative controls for 72 hrs. As shown in Figure 2C, overexpression of RCAN1 markedly decreased cell viability (Figure 2C, *p*<0.05) compared with negative controls in U251 and T98G cells. And cell colony formation assay showed that RCAN1 overexpression reduced colony forming efficiency compared with negative controls in U251 and T98G cells (5.32±0.23% compared with 10.54±0.19% of control in U251, and 3.33±0.31% compared with 7.74±0.43% of control in T98G, *p*<0.05, Figure 2D and 2E).

**Figure 3:**
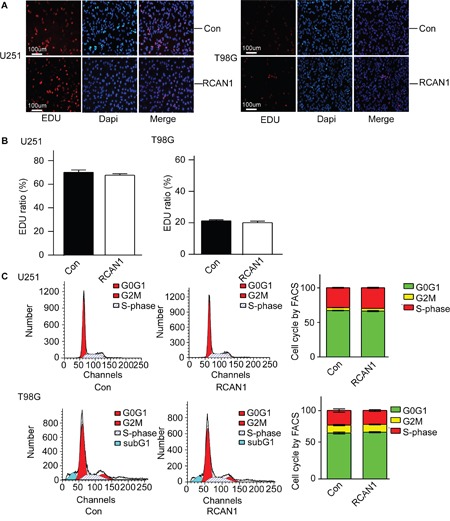
RCAN1 overexpression had no effect on cell cycle arrest U251 and T98G cells were infected with lentivirus expressing RCAN1 (RCAN1) or negative control (Control) for 72 hrs. **A**. Proliferative cells shown by EDU staining (red) and nuclei were counterstained with DAPI (blue). Results were analyzed by a fluorescent microscope with ×100 magnification. Data represents mean ± S.E. (n = 3). **B**. Quantification of (A). Values represent means ± SE (n = 3),*p <0.05 by Student's *t* test. **C**. U251 and T98G cellswere stained with PI and analyzed for their DNA content using FACS Calibur. Values represent means ± SE (n = 3). The right lanes were quantification of (C). Values represent means ± SE (n = 3),*p <0.05 by Student's *t* test.

The decrease of cell viability detected by MTT assay and colony formation assay could be either due to increased cell death or decreased cell proliferation. To differentiate this, Edu staining was performed to evaluate the cell proliferation. The results showed that overexpression of RCAN1 do not have an important role in the proliferation of glioma cells (*p*>0.05, Figure 3A and 3B). Consistent with EdU cell proliferation assay, flow cytometry test of cell cycle also showed RCAN1 had no effect on cell cycle (*p*>0.05, Figure 3C). Sub G1 population was quantified in T98G cell lines. And the result showed that RCAN1 overexpression increased Sub G1 population compared with negative control (11.15 ±0.36% compared with 9.51±0.06 % of controls in T98G, *p*<0.05, Figure 3C).

### RCAN1 overexpression induced glioma cell apoptosis

**Figure 4:**
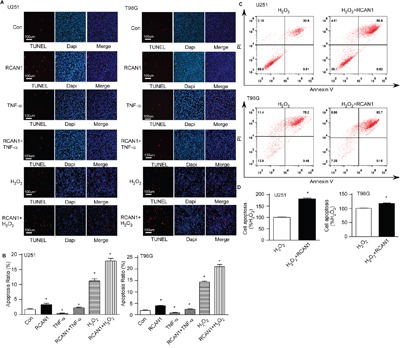
RCAN1 overexpression induced glioma cell lines apoptosis **A**. U251 and T98G cells were infected with lentivirus expressing RCAN1 (RCAN1) or negative control (Control) for 72 hrs. TUNEL staining was used to indicate cell apoptosis (red color) (*lane1* and *lane2*). Nuclei were counter stained with DAPI (blue color). Then the infected cells were further treated with 10μm H_2_O_2_ for 4 h (*lane5 and lane6*). About 20 ng/ml TNF-α was used to treat glioma cells infected with lentivirus for 3 min (*lane 3 and lane4*). Apoptotic cells were indicated by a magenta color, which corresponds to an overlap of red TUNEL and blue DAPI staining. The results were analyzed by a fluorescent microscope with ×100 magnification. **B**. Quantification of (A). Values represent means ± SE (n = 3), *p <0.05 by Student's *t* test. **C**. U251and T98G cells transfected with RCAN1expression plasmid, were stained with PI and Annexin V and analyzed by FACS to detect cell apoptosis. **D**. Quantification of (C). Values represent means ± SE (n = 3), *p <0.05 by Student's *t* test.

Since MTT assay showed RCAN1 decreased cell viability without affecting cell proliferation, we examined if RCAN1 affected glioma cell apoptosis. TUNEL staining was performed in glioma cells infected with lentivirus expressing RCAN1. Compared with the control, overexpression of RCAN1 renders more glioma cells death, a 3.33± 0.41% apoptosis ratio relative to 1.73 ± 0.27% of control by TUNEL staining in U251 cells, and a 3.98± 0.17% apoptosis ratio relative to 1.91 ± 0.24% of control by TUNEL staining in T98G cells (*p* < 0.05, Figure 4A and 4B, *lane 2 versus lane 1*). RCAN1 overexpression also exacerbated the toxicity of H_2_O_2_ on U251 cells, 17.87± 0.88% compared with 11.71± 0.79% in control (*p* < 0.05, Figure 4A and 4B, *lane 6 versus lane 5*). And RCAN1 overexpression also exacerbated the toxicity of H_2_O_2_ on T98G cells, 21.06 ± 0.93% compared with 14.34 ± 0.41% in control (*p* < 0.05, Figure 4A and 4B, *lane 6 versus lane 5*). Tumor necrosis factor TNFα -NF-κB activator could decreased the apoptosis ratio to 0.38±0.12% relative to 1.73 ± 0.27% of control in U251 cells (*p* < 0.005, Figure 4A and 4B, *lane 3 versus lane 1*), or 0.8567±0.234% relative to 1.91 ± 0.24% of control in T98G cells (*p* < 0.05, Figure 4A and 4B, *lane 3 versus lane 1*). And RCAN1 overexpression exacerbated the glioma cell apoptosis after TNFα treatment (*p* < 0.005, Figure 4A and 4B, *lane 3 versus lane 4*). All data suggested that RCAN1 induced apoptosis through NF-κB signaling pathway.

U251 and T98G cells were transfected with RCAN1 overexpression plasmid or its control vector. 48 hours after transfection, cells were exposed to 10 μM H_2_O_2_ treatment for 4 hours. The flow cytometry of Annexin V also showed that RCAN1 induced apoptosis to 181.50±4.04% in U251 (*p*<0.001) and 118.40±1.62 % (*p*<0.05) in T98G (Figure 4C and 4D). Taken together, our data showed that RCAN1 overexpression can suppress glioma cells viability through inducing apoptosis rather than arrest of cell cycle.

### RCAN1 suppressed viability of glioma cells through inhibiting NF-κB signaling pathway

**Figure 5:**
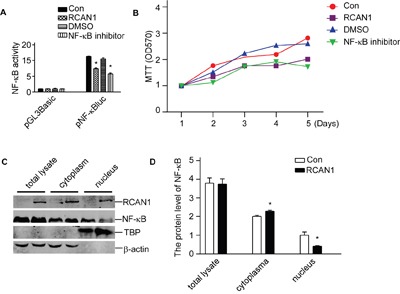
RCAN1 suppressed viability of glioma cells through inhibiting NF-κB signaling pathway U251 and T98G cells were infected with lentivirus expressing RCAN1 (RCAN1) or negative control (Control) for 72 hrs. **A**. RCAN1 reduced the luciferase activity controlled by NF-κB. These cells infected by lentivirus expressing RCAN1were transfected with pNF-κBLuc. Renilla luciferase activity was used to normalize transfection efficiency. Dual luciferase assay was performed 24 h after transfection. Data were calculated from 3 experiments. Values represent the means ± S.E. (n = 3), *p <0.05 by Student's *t* test. **B**. Cells viability was measured by MTT assay. The values represent the means ± S.E. (n = 3), *p*<0.05 by two-way ANOVA. **C**. Nuclear proteins were extracted. NF-κB/p65 protein levels were detected by anti-p65 antibody. RCAN1 protein was detected by anti-RCAN1 antibody. β-actin was used as loading controls for cytosolfractions. TBP detected by anti-TBP was used as loading controls for nuclear proteins. **D**. Quantification of (C). Values represent the means ± S.E. (n = 3), *p <0.05 by Student's *t* test.

NF-κB is the major survival factor in cells and constitutively activated NF-κB has been observed in various gliomas. To investigate if RCAN1 suppresses glioma viability by inhibiting NF-κB signaling pathway, we infected T98G cells by lentivirus expressing RCAN1 for 72hrs. Then these infected cells were transfected by reporter pNF-κBLuc plasmid. Dual luciferase assay showed that overexpression of RCAN1 decreased NF-κB luciferase activity compared with controls (11.33±0.07 compared with 7.43±0.20 relative luciferase units, *p*<0.05, Figure 5A). The potent NF-κB activation inhibitor (10 nM) (Calbiochem, #481412, Beijing, China) was used as positive controls (*p*<0.05, Figure 5A). To investigate if the NF-κB inhibition by RCAN1 will affect cell viability, T98G cells were infected with lentivirus expressing RCAN1. RCAN1 markedly decreased cell viability (*p*<0.05, Figure 5B).

NF-κB activity is mainly controlled by its nuclear translocation. To examine if RCAN1 affected NF-κB translocation, nuclear proteins were extracted, and NF-κB/p65 western blot showed that RCAN1 do not decrease the total NF-κB/p65 level in total lysate (*p*>0.05, Figure 5C and 5D). But RCAN1 significantly decreased the NF-κB/p65 level in the nucleus to 41.24±2.14% of controls (*p*<0.05, Figure 5C and 5D). The increase of NF-κB/p65 level in cytoplasm by RCAN1 compared with the control (2.00 ±0.07compared with 2.29±0.06, *p*<0.05, Figure 5C and 5D) corresponded to the decrease in nucleus fraction, indicating the decrease of NF-κB translocation in RCAN1 infected cells.

**Figure 6:**
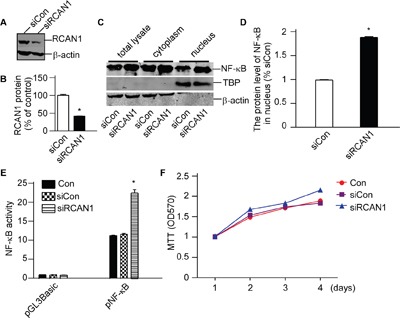
RCAN1 suppressed viability of glioma cells through inhibiting NF-κB signaling pathway T98G cells were transfected with pSuper-based shRNA plasmid (siRCAN1) or negative control (siCon) for 48 hrs. **A**. The knockdown effect of psiRCAN1 was verified in protein level by Western blot. RCAN1 was detected with anti-RCAN1 antibody. β-actin was used as the internal control. The values represented the means ±S.E. (n=3).*p <0.05 by Student's *t* test. **B**. Quantification of (A). Values represent the means ± S.E. (n = 3), *p <0.05 by Student's *t* test. **C**. Nuclear proteins were extracted. NF-κB/p65 protein levels were detected by anti-p65 antibody. β-actin was used as loading controls for cytosolfractions. TBP detected by anti-TBP was used as loading controls for nuclear proteins. **D**. Quantification of (C). Values represent the means ± S.E. (n = 3),*p <0.05 by Student's *t* test. **E**. RCAN1 knockdown increased the luciferase activity controlled by NF-κB. These cells were cotransfected with siRCAN1 and pNF-κBLuc. Renilla luciferase activity was used to normalize transfection efficiency. Dual luciferase assay was performed 24 h aftertransfection. Data were calculated from 3 experiments. Values represent the means ± S.E. (n = 3),*p <0.05 by Student's *t* test. **F**. Cell viability was measured by MTT assay. The values represent the means ± S.E. (n = 3), *p*<0.05 by two-way ANOVA.

To further confirm the effect of RCAN1 in NF-κB signaling, a pSuper-based shRNA plasmid (psiRCAN1) was used to knockdown expression of RCAN1. The knock-down efficiency of psiRCAN1 was detected by western blot. Silencing plasmid pSuper was performed as control. Western blot confirmed that RCAN1 expression was decreased with psiRCAN1 to 41.08±0.46% of controls (*p*<0.05, Figure 6A). Knockdown of RCAN1 could markedly increase the NF-κB/p65 levels in the nucleus to 188.30±1.40% of controls (*p*<0.05, Figure 6C and 6D). And knockdown expression of RCAN1 had significantly higher NF-κB luciferase activity compared with controls (22.45±0.86 compared with 11.33 ±0.07 relative luciferase units, *p*<0.05, Figure 6E) indicating that knockdown expression of RCAN1 can activate NF-κB signaling pathway. Meanwhile, knockdown expression of RCAN1 markedly increased cell viability (*p*<0.001, Figure 6F) compared with control. These results clearly indicate that RCAN1 can inhibit the transcriptional activity of NF-κB to suppress the viability of glioma cells.

## DISCUSSION

The results of the present study demonstrated that overexpression of RCAN1significantly inhibited NF-κB signaling pathway and induced apoptotic cell death in glioma cells. RCAN1 knockdown dramatically activated NF-κB signaling pathway and promoted the viability of glioma cells. Therefore, RCAN1 could be a novel and valuable anti-glioma target.

A series of prospective studies has shown that Alzheimer disease (AD) is associated with a reduced risk of cancer [16–18]. However, the mechanisms underlying this association remain unknown. We guessed that some protein highly expressed in AD can decline the progress of cancers. Because Down Syndrome (DS) patients almost universally develop early-onset AD [19], genes overexpressed by trisomy 21 provide a promising window into the pathogenesis of both DS-related AD and sporadic AD. Regulator of calcineurin 1 (RCAN1) is located on chromosome 21 in the region of 21q22.12. RCAN1 has been found in DS brains [20] and AD brains [21, 22]. NF-κB signaling pathway was a classical signaling pathway involved in many physiological and pathological processes. For example, NF-κB was participated in the pathogenesis of cerebral aneurysm [23], and activation of NF-κB may be one of the initiating factors contributing to the occurrence and development of cerebral aneurysm [24, 25]. Meanwhile, NF-κB signaling pathway has been considered as a therapeutic target in cancer because of its role in carcinogenesis [14]. Here, we primarily focused on the inhibition of NF-κB activity in tumor cells. And recent study has verified the RCAN1 can suppress cancer growth [4, 7, 26] by inhibiting NF-κB signaling pathway. In our study, we verified NF-κB signaling pathway was activated in glioma tissues. Meanwhile, we confirmed RCAN1 expression can inhibit NF-κB signaling pathway in glioma cells by attenuating NF-κB protein nuclear translocation, and resulted in suppressing the growth of glioma cells. NF-κB signaling pathway is a classical signaling pathway in cancer, which can regulate cell proliferation or apoptosis. In this study, we confirmed that the suppressing glioma cell growth was closely related to the pro-apoptosis effect, not inhibiting proliferation by the arrest of cell cycle.

So we want to explicit if RCAN1 can suppress the growth of glioma and becomes a new therapeutic target. Our study here identified RCAN1 can suppress glioma growth as an inhibitor of NF-κB signaling pathway [27]. The underlying mechanism is that RCAN1 can inhibit the nuclear translocation of NF-κB protein then affect the activity of NF-κB signaling pathway. And knockdown expression of RCAN1 could activate NF-κB signaling and promote the growth of glioma cells, clearly indicating that RCAN1 can inhibit the transcriptional activity of NF-κB to suppress the growth of glioma cells. Sustained activation of NF-κB is prevalent in cell lines and tumor tissue specimens and contributes to malignant progression and therapeutic resistance in most of the major forms of human cancer [28]. Since NF-κB is a popular cancer drug target, the identification of RCAN1 as an NF-κB inhibitor provides a potential treatment for cancer in which NF-κB signaling is aberrantly activated.

## MATERIALS AND METHODS

### Patients and samples

We analyzed the archival records from the collection of glioma files of Department of Neurosurgery, Beijing Tian Tan Hospital, Capital Medical University, Beijing, China. All the study protocols involved with patients and healthy donors were approved by the Medical Ethics Committee of BeijingTian Tan Hospital, Capital Medical University, Beijing, China, and written informed consents were obtained from all patients and healthy donors.

### Western blot analysis

Cell lysates were extracted with Ripa lysis buffer (50mM Tris, pH 7.4, 150mM NaCl, 1% Triton X-100,1% sodium deoxycholate,0.1% SDS, sodium orthovanadate, sodium fluoride, EDTA and leupeptin) supplemented with Roche Complete protease inhibitor cocktail. The protein concentration in the supernatant was measured with a bicinchoninic acid (BCA) assay kit (Thermo). SDS-PAGE was then performed using a 10% or 12% gel to isolate the proteins. After electrophoresis, proteins were transferred onto nitrocellulose filter (NC) membrane (Whatman) and hybridized with primary antibodies. The primary antibodies anti-TBP (T1827) and anti-actin mAb (A2228) were from Sigma-Aldrich (Shanghai, China). Anti-p65 antibody anti-IKKα, and anti-IKKβ were from Cell Signaling Technology (#9936, Danvers, MA, USA). The Page Ruler Prestained Protein Ladder from Thermo Scientific (#26616, Waltham, MA, USA) was used to indicate the target protein. Detection and quantification were performed with the Li-cor Odyssey imaging system and its software (Lincoln, NE, USA). 1:1000 overnight at 4°C. The secondary antibodies (IRdey800/680 goat anti mouse, or IRdey800/680 goat anti rabbit, Li-Cor) were all used at a dilution of 1:10000.

### Cell culture, transfection and drug treatment

U251 and T98G cells were cultured in Dulbecco's modified Eagle's medium (DMEM) containing 10% FBS, 1 mM sodium pyruvate, 2 mM L-glutamine, 50 unit/ml penicillin G sodium and 50 μg/ml streptomycin sulfate (Invitrogen). All cells were maintained in a 37°C incubator containing 5% CO2. All transfections were carried out with lipofectamineTM2000 transfection reagent (Invitrogen) according to the manufacturer's instructions. The potent NF-κB activation inhibitor (Calbiochem, #481412, Beijing, China) was dissolved in DMSO to 100mM and diluted with complete cell culture medium to the final concentration of 10uM. About 20 ng/ml TNF-α was used as NFκB activator. TNF-α was purchasedfrom Life Technologies (#PSC3015, Waltham, MA, USA).

### Real-time quantitative RT-PCRmRNA

Total RNA was isolated from brains using TRIzol reagent (Invitrogen). The mRNA of NF-κB was quantified by TOYOBO R SYBR Green gene Expression Analysis kit (TOYOBO, Japan). Primers for real-time quantitative PCR were as follows: human NF-κB, forward, 5’-ATGGCAGACGATGATCCCTAC-3’, and reverse, 5’-TGTTGACAGTGGTATTTCTGGTG-3’; RCAN1, forward, 5’-CGACTGGAGCTTCATTGACT-3’, and reverse, 5’-CCCAAGCTTTCCGCTGAGGTGGATCGGCGTGTA-3’;human β-actin, forward, 5’-GACAGGATGCAGAAGGAGAT-3’, and reverse, 5’-TGATCCACATCTGCTGGAAGGT-3’.

### Plasmid construction and lentivirus packaging

The cDNA of RCAN1 was generated as previously described [29]. Then the production was PCR amplified and cloned into lentivirus vector pWPXL. The lentivirus expression vector was co-transfected with psPAX2 and pMD2.G into HEK293T cells for lentivirus production. Lentivirus was harvested from the culture media 48 hours after transfection and precipitated with PEG8000. The titer of lentivirus produced is about 10^7^pfu/ml. pSuper-based shRNA plasmid (si-RCAN1) was generated as previously described [7].

### MTT assay and cell clone formation assay

Cell viability was detected with MTT and Colony formation assay. Briefly, 10,000 cells were seeded into 96-well culture plates and infected with lentivirus. When measuring, cells were incubated with 10 μL MTT (Cat: ST316, Beyotime, Shanghai, China) (5 mg/mL) at 37°C for 4 hrs, and then 100 μL 10% SDS(PH 4.0) was added to each well and incubated over night. The absorbance was measured at 570 nm (ref: 670 nm) with the Thermo Scientific microplate reader Varioskan Flash (Waltham, MA, USA). Each experiment was repeated at least three times. Cell colony formation assay: Tumor cells were digested by 0.25% trypsin/0.02% EDTA solution at the logarithmic phase to make a single-cell suspension with culture medium. Then, a cell counting chamber was used to calculate the number of cells in a 10 μl single-cell suspension under an inverted microscope. The cells were then delivered into six-well culture plates containing a sterile glass cover-slip in each well, with 2,000 cells added to each well. The medium was refreshed every 3 days until cell clones could be observed with the naked eye.

### Cell cycle analysis

Cells were harvested and washed twice in PBS, then fixed in 75% ethanol over night at 4°C. After washed in cold PBS three times, cells were resuspended in 1 mL PBS with 40 μg PI and 100 μg RNase A (Sigma-Aldrich, St Louis, MO) and incubated for 30 min at 37°C. Samples were then analyzed by BD FACSAria III cell sorter (San Jose, CA, USA).

### Dual luciferase assay, nucleus extraction, and ELISA assay

Luciferase activity was determined as previously described [30]. Nuclear extraction was performed using Nuclear Extraction Kit (Millipore, Beijing, China) following themanufacturer's instructions. ELISA assay was performed using the Human-NFκB p65 ELISA kit (Antibodies, USA) following the manufacturer's instructions.

### EDU and TUNEL staining

EdU staining was performed using EdU Kit (Ribobio, Beijing, China) following the manufacturer's instructions. For TUNEL staining, cells were fixed in 4% paraformaldehyde for 30 min and permeabilized with 0.1% TritonX-100 for 10 min. TUNELs taining was performed using the Roche-In Situ Cell Death Detection Kit according to themanufacturers’ instructions. Results were analyzed by fluorescence microscopy (LeicaDMI4000B). And cell nucleus was stained with 1 μg/ml DAPI (D9542; Sigma-Aldrich, USA) at 25°C for 10 min.

### Apoptosis assay

The flowcytometry was performed using PI and Annexin staining kit (BestBio, Cat: BB-4101-3), detected by BD FACSAria III cell sorter (San Jose, CA, USA) and analyzed using FlowJo Software 10.0.

### Data analysis

All experiments were repeated three to five times. In figures one representative picture was shown; quantifications were from three or five independent experiments. The values represent the means ± SEM. The data were evaluated for statistical significance with Student's *t* test analysis and two-way ANOVA.

## References

[1] Musicco M, Adorni F, Di Santo S, Prinelli F, Pettenati C, Caltagirone C, Palmer K, Russo A (2013). Inverse occurrence of cancer and Alzheimer disease: a population-based incidence study. Neurology.

[2] Sun X, Wu Y, Chen B, Zhang Z, Zhou W, Tong Y, Yuan J, Xia K, Gronemeyer H, Flavell RA, Song W (2011). Regulator of calcineurin 1 (RCAN1) facilitates neuronal apoptosis through caspase-3 activation. J Biol Chem.

[3] Minami T, Miura M, Aird WC, Kodama T (2006). Thrombin-induced autoinhibitory factor, Down syndrome critical region-1, attenuates NFAT-dependent vascular cell adhesion molecule-1 expression and inflammation in the endothelium. J Biol Chem.

[4] Baek KH, Zaslavsky A, Lynch RC, Britt C, Okada Y, Siarey RJ, Lensch MW, Park IH, Yoon SS, Minami T, Korenberg JR, Folkman J, Daley GQ (2009). Down's syndrome suppression of tumour growth and the role of the calcineurin inhibitor DSCR1. Nature.

[5] Shin J, Lee JC, Baek KH (2014). A single extra copy of Dscr1 improves survival of mice developing spontaneous lung tumors through suppression of tumor angiogenesis. Cancer Lett.

[6] Zheng L, Liu H, Wang P, Song W, Sun X (2014). Regulator of calcineurin 1 gene transcription is regulated by nuclear factor-kappaB. Curr Alzheimer Res.

[7] Liu C, Zheng L, Wang H, Ran X, Liu H, Sun X (2015). The RCAN1 inhibits NF-kappaB and suppresses lymphoma growth in mice. Cell Death Dis.

[8] Ohgaki H (2009). Epidemiology of brain tumors. Methods Mol Biol.

[9] Stupp R, Mason WP, van den Bent MJ, Weller M, Fisher B, Taphoorn MJ, Belanger K, Brandes AA, Marosi C, Bogdahn U, Curschmann J, Janzer RC, Ludwin SK (2005). Radiotherapy plus concomitant and adjuvant temozolomide for glioblastoma. N Engl J Med.

[10] Wen PY, Kesari S (2008). Malignant gliomas in adults. N Engl J Med.

[11] Phillips HS, Kharbanda S, Chen R, Forrest WF, Soriano RH, Wu TD, Misra A, Nigro JM, Colman H, Soroceanu L, Williams PM, Modrusan Z, Feuerstein BG (2006). Molecular subclasses of high-grade glioma predict prognosis, delineate a pattern of disease progression, and resemble stages in neurogenesis. Cancer Cell.

[12] Labussiere M, Wang XW, Idbaih A, Ducray F, Sanson M (2010). Prognostic markers in gliomas. Future Oncol.

[13] Gerondakis S, Grossmann M, Nakamura Y, Pohl T, Grumont R (1999). Genetic approaches in mice to understand Rel/NF-kappaB and IkappaB function: transgenics and knockouts. Oncogene.

[14] Ben-Neriah Y, Karin M (2011). Inflammation meets cancer, with NF-kappaB as the matchmaker. Nat Immunol.

[15] Pacifico F, Leonardi A (2006). NF-kappaB in solid tumors. Biochem Pharmacol.

[16] Burke WJ (2010). Cancer linked to Alzheimer disease but not vascular dementia. Neurology.

[17] Romero JP, Benito-Leon J, Louis ED, Bermejo-Pareja F (2014). Alzheimer's disease is associated with decreased risk of cancer-specific mortality: a prospective study (NEDICES). J Alzheimers Dis.

[18] Benito-Leon J, Romero JP, Louis ED, Bermejo-Pareja F (2014). Faster cognitive decline in elders without dementia and decreased risk of cancer mortality. NEDICES Study. Neurology.

[19] Lott IT, Head E (2001). Down syndrome and Alzheimer's disease: a link between development and aging. Ment Retard Dev Disabil Res Rev.

[20] Perluigi M, Di Domenico F, Buttterfield DA (2014). Unraveling the complexity of neurodegeneration in brains of subjects with Down syndrome: insights from proteomics. Proteomics Clin Appl.

[21] Cook CN, Hejna MJ, Magnuson DJ, Lee JM (2005). Expression of calcipressin1, an inhibitor of the phosphatase calcineurin, is altered with aging and Alzheimer's disease. J Alzheimers Dis.

[22] Wong H, Levenga J, Cain P, Rothermel B, Klann E, Hoeffer C (2015). RCAN1 overexpression promotes age-dependent mitochondrial dysregulation related to neurodegeneration in Alzheimer's disease. Acta Neuropathol.

[23] Fan XJ, Zhao HD, Yu G, Zhong XL, Yao H, Yang QD (2015). Role of inflammatory responses in the pathogenesis of human cerebral aneurysm. Genet Mol Res.

[24] Liu Y, Zhang Y, Dai D, Xu Z (2014). Expression of NF-kappaB, MCP-1 and MMP-9 in a Cerebral Aneurysm Rabbit Model. Can J Neurol Sci.

[25] Cheng WT, Wang N (2013). Correlation between MMP-2 and NF-kappa B expression of intracranial aneurysm. Asian Pac J Trop Med.

[26] Saenz GJ, Hovanessian R, Gisis AD, Medh RD (2015). Glucocorticoid-mediated co-regulation of RCAN1-1, E4BP4 and BIM in human leukemia cells susceptible to apoptosis. Biochem Biophys Res Commun.

[27] Wu Y, Song W (2013). Regulation of RCAN1 translation and its role in oxidative stress-induced apoptosis. FASEB J.

[28] Van Waes C (2007). Nuclear factor-kappaB in development, prevention, and therapy of cancer. Clin Cancer Res.

[29] Liu H, Wang P, Song W, Sun X (2009). Degradation of regulator of calcineurin 1 (RCAN1) is mediated by both chaperone-mediated autophagy and ubiquitin proteasome pathways. FASEB J.

[30] Sun X, Wang Y, Qing H, Christensen MA, Liu Y, Zhou W, Tong Y, Xiao C, Huang Y, Zhang S, Liu X, Song W (2005). Distinct transcriptional regulation and function of the human BACE2 and BACE1 genes. FASEB J.

